# Piglets' acute responses to procaine-based local anesthetic injection and surgical castration: Effects of two volumes of anesthetic

**DOI:** 10.3389/fpain.2022.943138

**Published:** 2022-08-09

**Authors:** Mathilde Coutant, Jens Malmkvist, Marianne Kaiser, Leslie Foldager, Mette S. Herskin

**Affiliations:** ^1^Department of Animal Science, Aarhus University, Tjele, Denmark; ^2^Bioinformatics Research Centre, Aarhus University, Aarhus, Denmark

**Keywords:** local anesthesia, castration, pig, pain, acute responses

## Abstract

Surgical castration of piglets is painful, but practiced routinely in commercial pig production. Procaine-based local anesthetics are used to mitigate piglet pain during castration in some countries. Yet, effects of the volume of anesthetic injected remain under-studied. The volume of drug administered may modulate the pain mitigating effect via variation in intra-testicular pressure at injection, potentially leading to pain or discomfort, as well as variation in the dose of active ingredient administered. The present study investigated the effects of injection with two volumes of a procaine-based local anesthetic, 0.3 vs. 0.5 mL per testis, on acute responses of 3–4 day old piglets. A total of 290 piglets were divided into 5 treatment groups: castration without anesthesia, castration after intra-testicular injection of 0.5 or 0.3 mL of drug per testis, and sham handling with one or two stays in a castration bench. Acute responses to drug injection, castration and sham handling were evaluated based on quantification of intra-procedural vocalizations and foreleg movements, as well as saliva cortisol concentrations before and after castration. Regardless of the volume, injection of anesthetic as well as castration led to significantly stronger responses than sham handling. Responses to the two drug volumes did not differ significantly, and responses to castration following injection of 0.3 mL did not differ from piglets castrated without anesthesia. All treatments, including sham handling, led to a significant increase in saliva cortisol, and no difference was found between anesthesia treatments and sham handling. Overall, the results suggested that injection of 0.5 mL led to better pain mitigation at castration compared to injection of 0.3 mL, but even when the local anesthetic was used, the combined procedures of injection and castration led to intra-procedural signs of pain and stress.

## Introduction

Surgical castration of piglets is performed routinely in commercial pig production in many countries. The removal of testes in newborn male piglets aims to prevent the development of boar taint, and reduce the prevalence of agonistic behaviors later in life (1, 2). Many studies have examined the procedure in terms of animal welfare. Without any pain mitigation, surgical castration leads to an increase in high frequency vocalizations during the procedure (3, 4), plasma cortisol concentrations in response to the procedure (5–8), and in-pen behavioral alterations up to days after the procedure (6, 9, 10) compared to sham handling. In order to address these welfare concerns, surgical castration of piglets is now commonly preceded by injections of local anesthetics. This pain mitigating strategy has been shown to reduce intra-procedural high frequency vocalizations (4, 11, 12), foreleg movements (11, 13–15), and to decrease the plasma cortisol response following castration (12, 16).

However, studies have shown that local anesthetics may mitigate, but not eliminate, the acute responses to castration, leaving doubts regarding the usefulness of the procedure (11). This has especially been questioned for procaine-based drugs, as these have a lower efficacy than drugs with e.g. lidocaine as active ingredient (17). Secondly, the use of needle-based injections and the required associated handling remain a concern, especially considering the limited knowledge of piglets' acute responses to injections (17, 18). Several aspects of this procedure, including method of injection, volume of drug injected, or time interval from injection to castration, have received limited scientific attention. It is also worth noting that, although herdsmen are allowed to perform the procedure in several countries including Denmark, only one study examined the response of piglets castrated following local anesthesia as performed in practice by herdsmen as opposed to experienced veterinarians or trained technicians (13).

We previously documented piglets' acute responses to different methods of injection of a procaine-based anesthetic and time intervals between injection and castration (19). This large study reported that piglets' responses to intra-testicular injection did not differ from a more complicated intra-funicular injection method, neither at injection nor at castration. Additionally, 5 to 10 min between injection and castration appeared superior among the tested intervals, using 0.5 mL of local anesthetic (equivalent to 10 mg procaine hydrochloride) per testis, in 3–4 day old piglets. However, one major finding was that the injection of local anesthetic in itself, regardless of the method, induced intra-procedural responses in piglets indicative of pain.

Piglets' acute responses to injection may be a consequence of penetration of the skin and testicular capsule by the needle, as well as increased intra-testicular pressure resulting from the injected liquid (20). Consequently, we hypothesized that a lower volume of anesthetic would lead to less acute responses in piglets during an intra-testicular injection. With no evidence that a higher dosage of procaine, corresponding to a higher volume of anesthetic injected, leads to an increased pain mitigating effect at castration (17, 21), it was further hypothesized that a lower volume would not compromise the pain mitigating effect at castration, though supporting data are scarce. It could also be argued that, if a lower dose was just as effective, this solution would present the benefits of being more cost-effective and reducing the risk of potential side-effects in piglets.

Based on the studies on piglet castration mentioned previously, it was hypothesized that a stronger acute response to the procedures, associated with pain, would result in a higher count of foreleg movements, a higher concentration of saliva cortisol, and a higher count of vocalizations, characterized by a stronger energy level and a higher distortion of the sound. In the absence of a gold standard to record pain in piglets, additional vocal indicators related to the duration of the calls were also measured.

Thus, this study investigated the impact of two volumes of a procaine-based local anesthetic injected intra-testicularly, 0.5 and 0.3 mL per testis, on piglets' acute responses as measured by vocalizations and counting of foreleg movements, both during injection and surgical castration, and by sampling of saliva for determination of cortisol 17 min after castration. The study was carried out as a field trial aiming to resemble commercial practice, and included control piglets castrated without any anesthesia, as well as piglets sham handled once or twice, to address the effects of handling itself.

## Materials and methods

### Animals

The experiment was carried out between June and September 2021 in a Danish conventional sow herd producing Landrace & Yorkshire x Duroc crossbred piglets. In the days after farrowing, sows were crated in farrowing pens measuring 3.1 × 2.8 m.

All experimental piglets were clinically healthy, free of overt anatomical malformations, weighed 0.96–2.47 kg and were 3–4 day old on the day of experimentation (with day 0 defined as the day of birth of the last piglet in a litter). Five male piglets were selected per litter, and randomly assigned to one of five treatments (Table 1), allowing all treatments to be present in each experimental litter. Piglets weighing less than 0.9 kg or more than 2.5 kg on the day of testing were not included in the study, to reduce the risk of improper fit in the castration bench. The piglets were administered a suspension of 45 mg toltrazuril and 200 mg gleptoferron (Forceris, 1.5 mL, Ceva Animal Health A/S, Libourne, France) on day 1 after farrowing, and were not ear tagged, tail docked, or teeth clipped before castration. After completion of the data collection and within 24 h after castration, piglets were administered an NSAID (NonSteroidal Anti-Inflammatory Drug) as analgesic by intramuscular injection of 1.5 mg meloxicam (Melovem, 0.3 mL, Dopharma, The Netherlands).

**Table 1:** Description of the five treatment groups involved in the study.

	**Treatment groups**				
	**V05**	**V03**	**CC**	**SH0**	**SH5**
Time interval (min)	5	5	-	-	5
Stays in the bench	2	2	1	1	2
Volume injected (mL)	0.5	0.3	-	-	-
Weight (kg)	1.71 ± 0.22	1.73 ± 0.30	1.68 ± 0.30	1.67 ± 0.29	1.66 ± 0.29
Duration of injection/ 1^st^ sham handling (s)	37 ± 6	35 ± 12	-	-	36 ± 3
Duration of castration/ 2^nd^ sham handling (s)	35 ± 5	38 ± 6	43 ± 10	37 ± 4	36 ± 3

*V05, castration 5 min after intra-testicular administration of 0.5 mL of drug per testis (n = 58); V03, castration 5 min after intra-testicular administration of 0.3 mL of drug per testis (n = 58); CC, castration without local anesthesia (control-castrated, n = 58); SH0, sham handling with one stay in the bench (n = 58); SH5, sham handling with two stays in the bench (n = 58). Weight and procedure duration are displayed as mean ± standard deviation (SD)*.

### Study design

On the day of castration, experimental piglets were weighed. Saliva samples were taken approximately 40 min before bringing the piglets to the testing area, a calm room outside of the farrowing room. All experimental piglets of a litter, plus one randomly selected littermate, were placed together in a plastic box (71.5 × 53.0 × 39.5 cm) layered with straw, underneath a heat lamp (averaging 20°C). Piglets were injected with local anesthetic, castrated or sham handled one by one. In-between procedures, piglets were returned to the heated box with their littermates. Immediately after castration or last sham handling, piglets were individually subjected to a 3 min social motivation test not reported in the present paper, and brought back to the sow in the farrowing pen. On average 17 min after castration or last sham handling, a second saliva sample was taken in the farrowing unit.

#### Sample size calculations

Power calculations were carried out for cortisol via simulations using a bivariate log-normal distribution in a mixed effects model setup, which was based on results from a pre-study (19). With a power of 80% at a significance level of 5%, a study of 5 piglets from each of 50 litters distributed randomly on 5 treatment groups, the following effects should be detectable: 1) 34% higher log(cortisol) concentration after injection with V03 compared to V05, and 2) 35–40% lower log(cortisol) concentration level after SH0 compared to SH5. In addition, differences between piglets being anesthetized prior to castration (V05, V03) compared to piglets castrated without anesthesia (CC) could always be detected, for all simulated scenarios.

#### Treatments

A total of 290 piglets were assigned to one of five treatments (Table 1): Castration without local anesthesia involving a single stay in the bench (control-castrated; CC), intra-testicular injection of 0.5 mL of local anesthetic per testis and subsequent castration after 5 min (V05), intra-testicular injection of 0.3 mL of local anesthetic per testis and subsequent castration after 5 min (V03), sham handling with no tissue damage inflicted with one stay (SH0) or two stays in the bench at a 5 min interval (SH5).

#### Procedures

All surgical and injection procedures were performed by the same experimenter, an experienced farm staff from Aarhus University, trained in accordance with standards from the Danish Veterinary and Food Administration [DVFA; (22)]. For anesthesia, 10 mg (V05) or 6 mg (V03) procaine hydrochloride (Procamidor® Vet., 0.5 mL or 0.3 mL, Richter Pharma AG, Wels, Austria) were injected in each testis. The product was administered using a 26G needle (0.45 mm × 12 mm, Sterican® Insulin needle, B Braun Medical SA, Barcelona, Spain) supplemented with a custom-made 5 mm plastic stopper, and fixed on an automatic syringe (Prima Tech® 0.5 mL in 0.1 mL increments). Needles were changed between each piglet. During all procedures, piglets were fixated while lying on their back in a commercially available castration bench (Unitron A/S, Kolding, Denmark). For the experimental purpose, the bench was modified to enable larger amplitudes of front leg movements, and more natural opening of the mouth during vocalizing. To further ensure a proper fit in the bench, considering the variation in piglets' body size, a soft material (5-mm yoga matt; Supplementary Figure 1) could be placed in the bench.

During injection with the local anesthetic, piglets were fixated in dorsal recumbency position in the castration bench, and testes were fixed carefully in the distal end of the scrotum. The right testis was fixed caudally between the thumb and index finger of the experimenter, applying a steady but low pressure during the fixation. The needle was inserted in the center of the right testis, in a dorsal direction at an angle of 90 degrees from a caudocranial view. A custom-made 5 mm plastic stopper was placed on each needle to ensure a standardized needle length of 7 mm. The anesthetic was injected slowly into the testis while gradually loosening the grip around the testis. The procedure was then repeated on the left testis. At castration, after fixation in the castration bench, a disposable scalpel (Scalpel no. 24, carbon steel sterile blade, Swann-Morton, Sheffield, England) was used to perform an incision (approximately 1 cm) through the scrotal skin and spermatic fasciae. The right testis was then gently pressed between the index and the thumb of the experimenter until fully outside of the scrotum. The testis was then carefully lifted vertically, and the spermatic cord cut a few millimeters below the testis using the scalpel. The incision was repeated on the left testis. A new scalpel was used for each piglet. Piglets sham handled were fixated in the castration bench for a duration of approximately 25 s (corresponding to the average duration of the procedures of local anesthesia and castration as assessed in a pilot study), during which they did not experience any tissue damage nor physical stimulation of the groin area. The specific time of day and duration (to the nearest second) was recorded for each procedure to the individual piglet.

### Ethical and other permits

The study was performed in compliance with the EU Directive 2010/63/EU for animal experiments, the Ministry of Food, Agriculture and Fisheries, and The Danish Veterinary and Food Administration under act 474 of 15. May 2014 and executive order 2028 of 14. December 2020. The study was approved as clinical trial by the Danish Medical Agency (reference numbers 021043561). All procedures were ethically evaluated and approved by the Danish Animal Experiments Inspectorate (approval numbers 2021-15-0201-00906).

### Data collection

#### Vocalizations

The intra-procedural vocal responses of the piglets were recorded during each procedure, using a microphone (Sennheiser E614, Sennheiser, Wennebostel, Germany) fixed 30 cm ahead of the piglet's snout, at the level of the head of the piglet. The microphone was connected to an amplifier (Audiobox USB® 96, PreSonus, Louisiana, USA) connected to a computer, from which recordings were manually started and stopped upon piglets' placement and removal from the castration bench. All vocal files were analyzed using Raven Pro 1.6 bioacoustics analysis software (Cornell Lab of Ornithology, Ithaca, New York, USA). An automatic detection and characterization of all piglet calls was developed using the band limited energy detector function in the Raven Pro software. This function allowed each intra-procedural call to be automatically detected based on a pre-set of parameters, and characterized in terms of number, duration, energy, and entropy. After running the automatic call detection, each procedural recording was manually checked to ensure that every call was properly selected, and to de-select surrounded noise or experimenters' voices wrongfully detected as a call. For all piglets, vocal characteristics of each procedure were then defined (Table 2) and analyzed. These procedures were performed by one person (MC), blinded to the experimental treatments.

**Table 2:** Description of the vocal parameters analyzed for each piglet during injection of local anesthetic, castration, or sham handling while in the castration bench.

**Parameter (unit)**	**Description**
Call proportion	Proportion of time spent vocalizing during the procedure, calculated as sum of call durations /procedure duration
Call per second (s^−1^)	Number of calls per s of the procedure.
Mean call duration (s)	Average duration of a call during the procedure, calculated as sum of call durations / number of calls.
Mean energy (dB)	Average energy, calculated as an average of the energy of each call during the procedure.
Max energy (dB)	Maximum value of energy recorded for all calls during the procedure.
Max power (dB)	Maximum power recorded for all calls during the procedure, relative to the specific recording set-up.
Aggregated entropy (kilobits)	Aggregated disorder for the procedure obtained by analyzing the energy distribution within each call. Higher entropy values correspond to greater disorder in the sound whereas a pure tone would have zero entropy (23).
Max entropy (kilobits)	Highest value of disorder recorded for all calls during the procedure.

#### Foreleg movements

Four distinct types of front leg movements were recorded during each procedure for each experimental piglet using a camera (GoPro HERO7 Black, GoPro, San Mateo, California, USA; 60 frames per sec, FPS) placed on a stand 30 cm to the right side of the castration bench, approximately 50 cm above the bench. Before this study, no validated, standardized method to quantify resistance existed. Initially, randomly chosen video clips were observed at low speed (5 FPS) to detect recurrent, identifiable movements. Four types of movements were selected and described: flexion, extension, kick, and blow (Table 3, Supplementary Figure 2). Two observers, blinded to the experimental treatments, were trained to recognize and count these behaviors, and practiced recording on approximately 100 random video clips, using the Behavioral Observation Research Interactive Software [BORIS; (24)]. Each video sequence was then analyzed, and the occurrence of each type of behavior was counted for each front leg in the interval between closing and opening of the castration bench. Movements that were too sudden to be categorized despite the low speed of video analysis were not counted. Reversely, movements performed relatively slow (duration > 1 s) were not considered as resistance and therefore not recorded. In addition, duration of blocking in the bench, corresponding to a leg being mechanically unable to move due to physical blocking, were also recorded.

**Table 3:** Description of the foreleg movements recorded during injection of local anesthetic, castration, or sham handling while in the castration bench.

**Category**	**Description**
Flexion	Piglet vertically bends his front leg, provoking a flexion of the elbow of at least 90 degrees.
Extension	Piglet fully extends his front leg while lowering its head in the bench. May be accompanied by trembling of the leg and/or by a subtle lift of the piglet's back.
Kick	Piglet's front leg performs a sudden upwards movement, changing from a flexion to a tense upwards position.
Blow	Piglet suddenly draws back his front leg forwards or backwards for at least half a bench length, from a normal upright position to an extended position, with little or no flexion of the elbow.
Leg blocked	Piglet's front leg is blocked in the bench cone, preventing movement.

*Each leg was scored separately*.

#### Saliva cortisol concentrations

For baseline, one saliva sample per piglet was collected approximately 40 (± 12, SD) min before the first procedure. For changes in saliva cortisol in response to the treatments, another sample was collected approximately 17 (± 1) min after castration. No standardized method currently exists to sample saliva in piglets as young as 3 day old. In this study, inspired by recent work in dogs (25), a cotton swab (Salivette®, Sarstedt, Aktiengesellschaft & Co., Numbrecht, Germany) was cut in pieces (~2.0 × 0.5 cm), soaked in concentrated apple juice (nectar from concentrated juice, min 60%, Rynkeby Foods A/S, Ringe, Denmark) for 1 h, and dried in an electric oven at 60 °C for 5 h. A pilot study revealed an increase in saliva production with this method compared to the use of a non pre-soaked piece of cotton. Similar results were obtained with soaking the cotton pieces in citric acid (fresh lemon juice), but after this method, saliva sampling seemed more aversive for the piglets, and the method was therefore abandoned. During sampling, the cotton swab was fixed at the end of a straight pean clamp, and gently introduced into the piglet's mouth, while the piglet was held in the experimenter's arms. The cotton was lightly rotated in the piglet's mouth for 30 to 45 s, with insistence around the salivary glands. This procedure was performed by one of four trained experimenters blinded to the experimental treatments. The samples were then placed in an experimental tube (provided as part of the Salivette®), labeled, and stored at −18 °C until cortisol concentration determination at our departmental laboratory. Samples were defrosted and centrifuged for 6 min at 1,000 × *g*. Concentrations of cortisol were determined using a direct enzyme immunoassay without extraction and previously validated for saliva (Arbor Assays, Cat. K003-H1W, Michigan, USA). In this method, the antiserum cross-reacts with cortisol and some cortisol metabolites, and values have to be interpreted as cortisol immunoreactivity. The intra-assay coefficient of variation was 3.7 and 5.6%, respectively for low and high control and the inter-assay variation was 7.2 and 9.8% for low and high control, respectively. The minimal detectable concentration was 45 pg/mL. The procedure outlined by the manufacturer was followed.

### Data handling

Two piglets were removed from the analysis as they appeared sick during testing. Both piglets showed an amelioration of their health status in the hours following the procedures. In addition, four piglets were excluded from the foreleg movement analysis due to technical issues with the video recording (3 for injection and 1 for castration). Malfunctioning and technical issues of the set-up recording vocalizations resulted in 61 missing files for injection of the local anesthetic/first sham handling, and 76 missing files for castration/second sham handling, leaving valid vocalization data from 166 piglets for local anesthesia/first sham handling and 209 piglets for castration/second sham handling (see Supplementary Figure 3).

### Statistical analysis

Vocalizations and foreleg movements were analyzed separately for injection and castration, but an additional analysis investigated the cumulative (i.e. summed over procedures) responses to injection plus castration. During the injection of the local anesthetic, number of stay in the bench had not yet any bearing, and thus three treatments were relevant: injection of 0.5 mL (V05) or 0.3 mL of drug (V03), and sham handling (SH; pooling SH0 and the first stay in the bench of SH5).

Vocalization parameters (Table 2) were log transformed if necessary (to obtain normality) and analyzed in linear mixed effects models with treatment (during injection: V05, V03, SH; during castration: V05, V03, CC, SH0, SH5) as the main explanatory variable, weight (range: 0.96–2.47 kg), age (3 or 4 days), hour of day when starting the procedure (decimal hour, range: 7.21–15.47) and duration of the procedure (range: 20–76 s) as covariates, and litter as a random effect. Rate of vocalizations, and other variables where duration of the procedure was an integrated part of the calculated response, were analyzed by similar models, but without duration of the procedure as covariate.

The count of each type of foreleg movements (Table 3) were summed to a total count of foreleg movements per piglet during each procedure. Total duration of observation was defined as the sum of observation time per leg, subtracting the duration of left leg and right leg occasionally being blocked in the bench. The sum of piglets' foreleg movements was analyzed by a negative binomial mixed effects model including treatment as main explanatory variable, weight, age and hour of day as covariates, logarithm of total observation duration (range: 1–205 s) as offset, and litter as a random effect.

Cortisol responses were log transformed and analyzed in a mixed model with the treatment as main explanatory variable, weight, age, hour of sampling (decimal hour, range: 6.85–15.72), and baseline cortisol concentration (range: 2,739–60,570 pg/mL) as covariates, and litter as random effect.

For all indicators, the initial models were reduced by stepwise removal of variables at P>0.10, however, keeping fixed effects of main interest (i.e. treatment group) in the model. In linear mixed effects models, Satterthwaite's approximation of the denominator degrees of freedom were used. Deviations from assumption of normality and variance homogeneity were monitored visually by plotting residuals at each step. Covariates with significant effects were maintained in the final models, but effects not reported, at the exception of weight. In case of the final model showing significant treatment effects (*P* ≤ 0.05), pairwise comparisons between treatments were performed with *p*-values adjusted for multiple comparisons using the Tukey-Kramer method (indicated by P_adj_). All calculations were performed using SAS 9.4 (SAS Institute Inc, Cary, North Carolina, USA). All data used in the statistical analysis are available in the dataset in Supplementary material. Descriptive measures are presented as averages ± standard error (SE).

## Results

### Vocalizations

In each section, results include descriptions of the effects recorded for each specific vocal indicator, and additionally, to ease understanding despite the large amount of data presented, a general picture of the patterns observed across indicators.

#### During injection of the local anesthetic

During injection of the local anesthetic, the treatments led to significantly different vocal responses. A significant treatment effect was observed in call proportion, mean energy, max energy, and aggregated entropy, with no difference between V05 and V03, and lower values recorded in SH (Table 4). In addition, call per second and max power were significantly different among treatments, with no difference between V05 and V03, but V05 differed from SH, while V03 did not. Mean call duration also differed among treatments, but, reversely to the preceding parameters, V03 differed from SH, while V05 did not. Max entropy was overall significantly impacted by the treatments, but none of the pairwise comparisons were significant after adjustment for multiple testing. In addition, piglet weight affected, or tended to affect, call proportion (F_1,130_ = 3.1, *P* = 0.082), mean call duration (F_1,149_ = 3.0, *P* = 0.083), and mean energy (F_1,141_ = 7.2, *P* = 0.008), with stronger responses observed in heavier piglets. Reversely, a lower max power response (F_1,125_ = 4.7, *P* = 0.031) was observed in heavier piglets.

**Table 4:** Averages (± SE) of vocal parameters recorded during injection of the local anesthetic.

	**V05**	**V03**	**SH**	**F test**	***P***
Call proportion	0.58 ± 0.03*^a^*	0.66 ± 0.03*^a^*	0.43 ± 0.03*^b^*	F_2, 126_ = 15.1	<0.001
Call per second (s^−1^)	0.82 ± 0.04*^a^*	0.75 ± 0.04*^ab^*	0.66 ± 0.03*^b^*	F_2, 163_ = 5.9	0.003
Mean call duration (s)	0.76 ± 0.05*^ab^*	0.98 ± 0.08*^a^*	0.62 ± 0.04^b^	F_2, 125_ = 10.9	<0.001
Mean energy (dB)	87.26 ± 14.98*^a^*	110.88 ± 15.4*^a^*	43.08 ± 10.77*^b^*	F_2, 124_ = 7.2	0.001
Max energy (dB)	306.66 ± 15.19*^a^*	281.52 ± 16.9*^a^*	223.89 ± 14.0*^b^*	F_2, 122_ = 6.8	0.002
Max power (dB)	−15.68 ± 1.13*^a^*	−18.38 ± 1.46*^ab^*	−21.61 ± 1.14*^b^*	F_2, 126_ = 4.7	0.011
Agg entropy (kilobits)	120.26 ± 4.47*^a^*	103.01 ± 5.01*^a^*	89.66 ± 4.45*^b^*	F_2, 162_ = 11.2	<0.001
Max entropy (kilobits)	5.78 ± 0.02	5.79 ± 0.03	5.71 ± 0.02	F_2, 126_ = 3.7	0.027

ab *Different letters within a row indicate significant difference between treatments, P_adj_ ≤ 0.05; V05, intra-testicular administration of 0.5 mL of drug per testis (n = 41); V03, intra-testicular administration of 0.3 mL of drug per testis (n = 42); SH, sham handling (n = 83)*.

Overall, the two volumes of local anesthetics did not lead to a significantly different response during the injection procedure, but the lowest volume, 0.3 mL per testis, resulted in vocal responses that did not differ significantly from those of sham handled piglets in terms of call per second and maximum power, while 0.5 mL did not differ from sham handled piglets in terms of mean call duration.

#### During castration

At castration, all indicators showed significant differences among treatments (Table 5). Only one indicator, call proportion, was significantly different between V05 and V03, with a greater proportion of calls recorded in piglets administered the lowest volume and thus dosage, V03. In addition, for this indicator, V03 did not differ significantly from CC. In 3 out 8 indicators, V05 showed values significantly lower than CC, while V03 did not (i.e. max energy and tendency for mean energy and max power). In half of the indicators, V03, V05 and CC did not differ significantly in their response (cf. Table 5), and, for aggregated entropy and call per second, these three treatments led to greater responses than SH0 and SH5. V05 led to vocal characteristics that did not differ significantly from those of SH0 and SH5 in 6 out of 8 indicators, while V03 did not differ significantly from the controls that were sham handled in 2 out of 8 indicators. CC did not differ significantly from SH0 and SH5 for 2 indicators, mean call duration and max entropy. Max power was impacted by piglet weight, with values decreasing with increasing weight (F_1,145_ = 8.1, *P* = 0.005).

**Table 5:** Averages (± SE) of vocal parameters recorded during castration.

	**V05**	**V03**	**CC**	**SH0**	**SH5**	**F test**	***P***
Call proportion	0.51 ± 0.03*^b^*	0.64 ± 0.03*^a^*	0.66 ± 0.02*^a^*	0.43 ± 0.04*^b^*	0.44 ± 0.04*^b^*	F_4, 165_ = 11.4	<0.001
Call per second (s^−1^)	0.86 ± 0.05*^a^*	0.87 ± 0.04*^a^*	0.89 ± 0.03*^a^*	0.67 ± 0.04*^b^*	0.65 ± 0.03*^b^*	F_4, 164_ = 9.8	<0.001
Mean call duration (s)	0.64 ± 0.04*^ab^*	0.82 ± 0.06*^a^*	0.79 ± 0.05*^a^*	0.61 ± 0.05*^b^*	0.70 ± 0.08*^ab^*	F_4, 164_ = 4.4	0.002
Mean energy (dB)	42.15 ± 12.91*^b^*	78.93 ± 12.00*^b^*	124.61 ± 13.40*^a^*	43.08 ± 14.83*^b^*	30.76 ± 14.13*^b^*	F_4, 163_ = 8.9	<0.001
Max energy (dB)	243.56 ± 17.55*^bc^*	287.69 ± 14.65*^ab^*	335.18 ± 9.52*^a^*	220.37 ± 21.18*^bc^*	218.08 ± 19.68*^c^*	F_4, 165_ = 9.7	<0.001
Max power (dB)	−20.78 ± 1.47*^b^*	−17.53 ± 1.18*^b^*	−13.82 ± 0.86*^a^*	−21.90 ± 1.71*^b^*	−22.75 ± 1.68*^b^*	F_4, 65_ = 6.7	<0.001
Agg entropy (kilobits)	115.12 ± 6.74*^a^*	125.08 ± 6.07*^a^*	151.79 ± 6.44*^a^*	89.26 ± 5.52*^b^*	89.17 ± 5.40*^b^*	F_4, 167_ = 14.4	<0.001
Max entropy (kilobits)	5.74 ± 0.02*^ab^*	5.82 ± 0.02*^a^*	5.77 ± 0.02*^ab^*	5.70 ± 0.03*^b^*	5.71 ± 0.04*^b^*	F_4, 166_ = 3.2	0.014

abc *Different letters within a row indicate significant differences between treatments, P_adj_ ≤ 0.05; V05, castration 5 min after intra-testicular administration of 0.5 mL of drug per testis (n = 41); V03, castration 5 min after intra-testicular administration of 0.3 mL of drug per testis (n = 42); CC, castration without local anesthesia (control-castrated, n = 43); SH0, sham handling with one stay in the bench (n = 42); SH5, sham handling with two stays in the bench (n = 41)*.

Overall, piglets administered 0.3 mL of local anesthetic per testis showed a vocal response at castration comparable or greater than the one of piglets administered 0.5 mL. Castration after injections of 0.3 mL of local anesthetic resulted in vocal characteristics that often did not differ significantly from those of piglets castrated without anesthesia, while castration after injections of 0.5 mL led to values that often did not differ significantly from sham handled piglets. In general, vocal responses of castrated piglets were significantly higher than those of sham handled piglets.

#### During injection and castration combined

**Figure 1:**
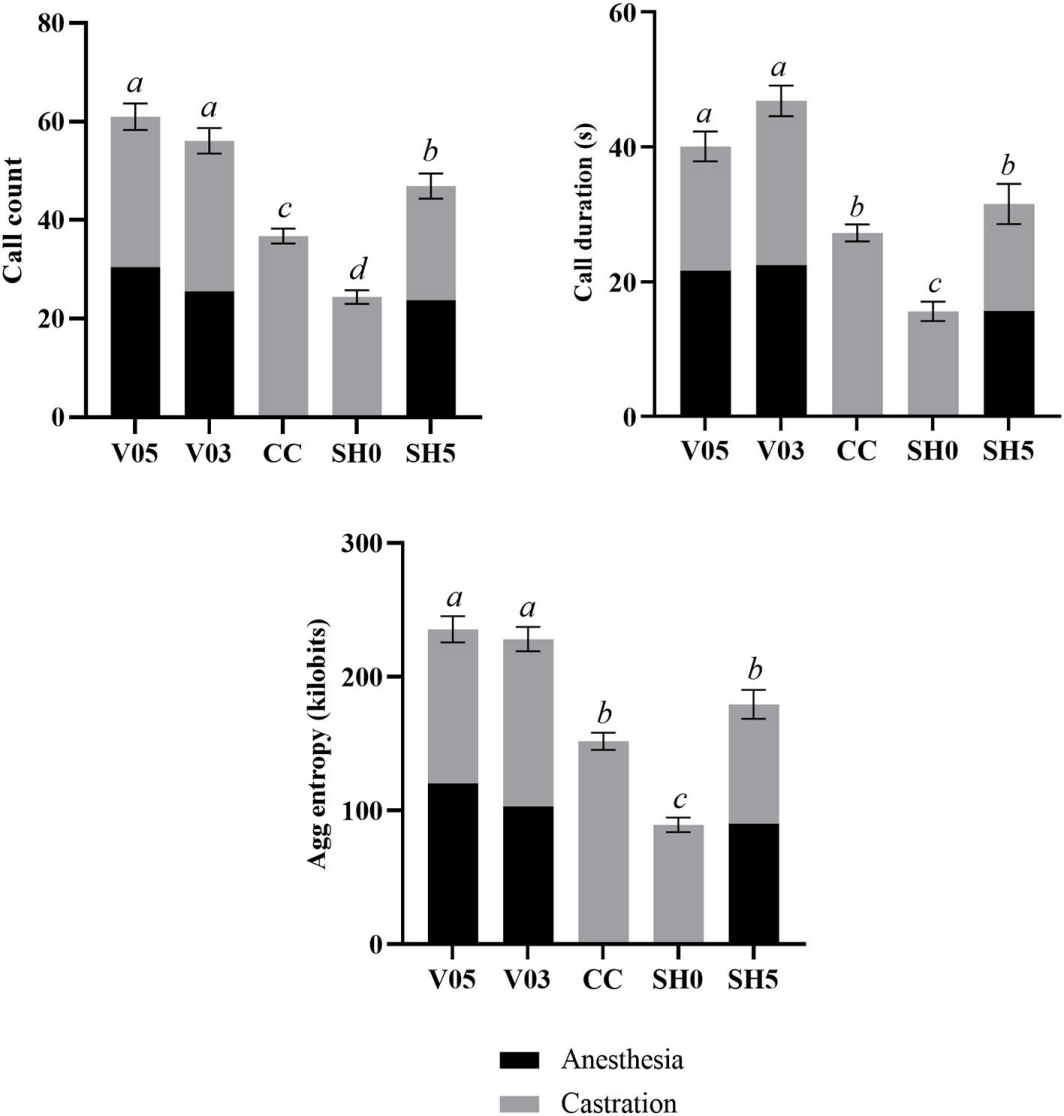
Combined call count, call duration and aggregated entropy parameters recorded during injection of local anesthetic and castration. Different letters indicate significant differences between treatments, P_adj_ ≤ 0.05; Error bars indicate standard errors of the cumulative averages. V05, intra-testicular injection of 0.5 mL of anesthetic per testis (*n* = 41); V03, intra-testicular injection of 0.3 mL of anesthetic per testis (*n* = 42); CC (control-castrated), castration without anesthesia (*n* = 43); SH0, sham handling with one stay in the bench (*n* = 42); SH5, sham handling with two stays in the bench (*n* = 41).

Analysis of cumulative counts, duration, and aggregated entropy of vocalizations (i.e. summed response per piglet during injection plus castration) showed significant differences among treatments (Table 6, Figure 1). For all three cumulative parameters, the two volumes (V05, V03) did not differ significantly, but the injection and the subsequent castration generated a total output of vocal responses significantly larger than the other three treatments, including castration without anesthesia. The total number of calls in sham handled piglets with two stays in the bench (SH5) outnumbered CC piglets (staying once in the bench). Sham handling with a single stay in the bench consistently led to the lowest vocal responses (Table 6).

**Table 6:** Averages (± SE) of vocal parameters recorded during combined injection of local anesthetic and castration.

	**V05**	**V03**	**CC**	**SH0**	**SH5**	**F test**	***P***
**Call count**	60.98 ± 2.68*^a^*	58.17 ± 2.57*^a^*	36.74 ± 1.52*^c^*	24.39 ± 1.40*^d^*	46.90 ± 2.53*^b^*	F_4, 165_ = 47.8	<0.001
**Total call duration (s)**	40.10 ± 2.21*^a^*	46.84 ± 2.26*^a^*	27.25 ± 1.25*^b^*	15.61 ± 1.44*^c^*	31.55 ± 2.98*^b^*	F_4, 165_ = 36.7	<0.001
**Agg entropy (kilobits)**	235.38 ± 9.72*^a^*	228.09 ± 9.04*^a^*	151.18 ± 6.44*^b^*	89.26 ± 5.52*^c^*	179.22 ± 10.82*^b^*	F_4, 165_ = 50.2	<0.001

abc *Different letter within a row indicate significant differences between treatments, P_adj_ ≤ 0.05; V05, castration 5 min after intra-testicular administration of 0.5 mL of drug per testis (n = 41); V03, castration 5 min after intra-testicular administration of 0.3 mL of drug per testis (n = 42); CC, castration without local anesthesia (control-castrated, n = 43); SH0, sham handling with one stay in the bench (n = 42); SH05, sham handling with two stays in the bench (n = 41)*.

### Foreleg movements

#### During injection of the local anesthetic

Piglets' leg response during the administration of the local anesthetic differed significantly among treatments (Table 7), with a higher number of foreleg movements per time unit recorded in piglets injected with the anesthetic, regardless of the volume, compared to sham handled piglets. The level of foreleg movements observed in piglets injected with 0.5 or 0.3 mL per testis did not differ significantly.

**Table 7:** Averages (± SE) of foreleg movements recorded during injection of local anesthetic and during castration.

	**V05**	**V03**	**CC**	**SH0**	**SH5**	**χ^2^ test**	***P***
Anesthesia	20.47 ± 1.82*^*a*^* (*n* = 55)	19.50 ± 2.15*^*a*^* (*n* = 56)	-	10.23 ± 1.05*^*b*^* (*n* = 115)		$\chi_{2}^{2}=24.0$	<0.001
Castration	24.09 ± 2.45*^*a*^* (*n* = 55)	32.71 ± 2.44*^*a*^* (*n* = 56)	39.66 ± 2.53*^*a*^* (*n* = 58)	11.45 ± 1.54*^*b*^* (*n* = 58)	11.12 ± 1.37*^*b*^* (*n* = 57)	$\chi_{4}^{2}=113.7$	<0.001
Anesthesia+ Castration	44.56 ± 3.64^a^ (*n* = 55)	51.30 ± 3.86^a^ (*n* = 56)	39.66 ± 2.53^a^ (*n* = 58)	11.45 ± 1.54^c^ (*n* = 58)	20.15 ± 2.17^b^ (*n* = 57)	$\chi_{4}^{2}=117.4$	<0.001

abc *Different letters within a row indicate significant differences between treatments, P_adj_ ≤ 0.05.; V05, castration 5 min after intra-testicular administration of 0.5 mL of drug per testis; V03, castration 5 min after intra-testicular administration of 0.3 mL of drug per testis; CC, castration without local anesthesia (control-castrated); SH0, sham handling with one stay in the bench; SH5, sham handling with two stays in the bench*.

#### During castration

At castration, the occurrence of foreleg movements differed significantly among treatments (Table 7). Sham handling treatments led to a significantly fewer movements than castration treatments, with no significant difference between castration after local anesthesia and castration without local anesthesia. The number of foreleg movements did not differ whether sham handled piglets stayed once or twice in the bench. During castration, piglets previously injected with 0.3 mL of anesthetic per testis showed over 30% more foreleg movements on average than piglets injected with 0.5 mL per testis. However, this difference did not reach statistical significance (Table 7).

#### During injection and castration combined

**Figure 2:**
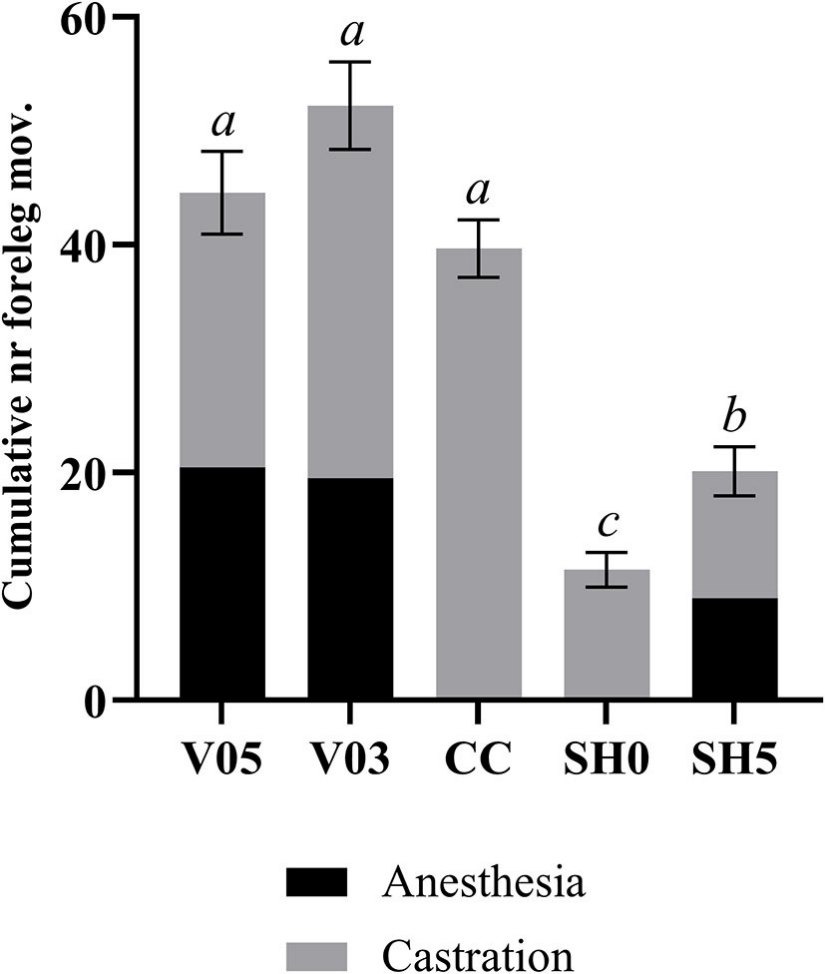
Combined number of foreleg movements recorded during injection of local anesthetic and castration. Different letters indicate significant differences between treatments, P_adj_ ≤ 0.05. Error bars indicate standard errors of the cumulative averages. V05, intra-testicular injection of 0.5 mL of anesthetic per testis (*n* = 55); V03, intra-testicular injection of 0.3 mL of anesthetic per testis (*n* = 56); CC (control-castrated), castration without anesthesia (*n* = 58); SH0, sham handling with one stay in the bench (*n* = 58); SH5, sham handling with two stays in the bench (*n* = 57).

Analysis of cumulated number of foreleg movements summed for injection plus castration showed significant differences among treatments (Table 7, Figure 2). Treatments involving castration did not differ significantly from each other, although piglets injected with 0.5 mL and 0.3 mL of anesthetic per testis showed, on average, 12% and 26% more foreleg movements than piglets castrated without anesthesia, respectively. All castration treatments resulted in significant higher levels of foreleg movements than sham handling, with a lower response in sham handled piglets placed once in the castration bench compared to piglets placed twice in the bench (Table 7).

### Saliva cortisol concentrations

As expected, baseline cortisol concentrations recorded 40 min before first procedure did not differ significantly among treatments (Table 8). An effect of weight was observed (F_1,137_ = 5.7, *P* = 0.018), with higher values of baseline cortisol recorded in lighter piglets. At 17 min post-castration/last sham handling, cortisol concentrations did not differ among treatments (Table 8).

**Table 8:** Averages (± SE) saliva cortisol concentrations (pg/mL) sampled at baseline and approx. 17 min post-castration/last sham handling.

	**V05**	**V03**	**CC**	**SH0**	**SH5**	**F test**	***P***
Baseline sample	10,071 ± 651 (*n* = 55)	10,784 ± 682 (*n* = 57)	10,047 ± 773 (*n* = 58)	12,165 ± 1,387 (*n* = 58)	10,697 ± 734 (*n* = 57)	F_4, 217_ = 0.8	0.501
Post- procedural sample	17,982 ± 1,259 (*n* = 55)	18,578 ± 967 (*n* = 57)	17,092 ± 1,045 (*n* = 56)	16,525 ± 1,014 (*n* = 58)	18,236 ± 1,133 (*n* = 57)	F_4, 217_ = 1.7	0.158

*V05, castration 5 min after intra-testicular administration of 0.5 mL of drug per testis; V03, castration 5 min after intra-testicular administration of 0.3 mL of drug per testis; CC, castration without local anesthesia (control-castrated); SH0, sham handling with one stay in the bench; SH5, sham handling with two stays in the bench*.

## Discussion

The present study investigated the effects of injection of two volumes of the same concentration of a procaine-based local anesthetic, 0.5 and 0.3 mL per testis, on piglets' acute responses as measured by their vocalizations and front leg movements during injection and castration, as well as post-procedural saliva cortisol concentrations. In contrast to our expectations, the results showed no significant difference in acute responses to intra-testicular injections with the two volumes of the drug. However, at castration, vocal responses of piglets injected with 0.3 mL of local anesthetic per testis often did not differ from responses of piglets castrated without prior local anesthesia, indicating a poor efficacy to mitigate castration pain.

Overall, the results showed that injection of a procaine-based local anesthetic may limit acute responses at castration as measured by vocalization characteristics. This result is in line with previous findings (15, 17). We also previously reported reduced acute responses in piglets castrated 5 min after intra-testicular injection of 0.5 mL of the same local anesthetic, compared to piglets castrated without local anesthesia, and, for both treatments, greater responses than sham handled piglets were found during injection and castration (19). Although not reaching significance (after adjustment for multiple testing), a similar pattern was observed in terms of leg movements in the present study, with almost 40% less foreleg movements recorded in piglets castrated after injection of 0.5 mL of drug per testis compared to piglets castrated without anesthesia. It is worth noting that the blinding of the observers recording the count of leg movements was partial, as the nature of the procedure (castration, injection or sham handling) was visible on the video. Considering the relative objectivity of our recording method as compared to for instance a scoring approach, it is unlikely that this limitation affected the results. However, the general methodology for this indicator could be further refined by cropping the rump area off the video prior to analysis, although this editing could make certain large amplitude movements harder to detect.

So far, only one study has investigated the effects of the dosage of procaine and lidocaine on piglets' responses to castration as measured by foreleg movements and intensity of vocalizations, using comparable injection methods. While the results showed that piglets' responses were negatively correlated to the volume of lidocaine administered, the same pattern was not detected for procaine, with no significant difference reported among four doses (10 mg, 20 mg, 30 mg or 40 mg per piglet) tested in a relatively small set-up with 32 piglets of 4 to 6 days of age per treatment group (21). The study did not specify whether the drug volume differed among dose treatments or not, so confounding effects cannot be excluded. Another study compared acute responses to injection of 0.3 or 0.5 mL of a procaine-based drug and subsequent castration in 3 to 7 day old piglets (17). The results did not show a significant difference in vocalizations or level of foreleg movements between the two drug volumes, neither during injection nor castration. It is worth noting, though, that the design included two different injection methods for the two volumes (intra-testicular for 0.3 mL and intra-funicular for 0.5 mL), potentially confounding the reported effects. Our results are somewhat in line with these previous findings, with no difference between the two volumes observed, neither during the injection nor during castration. However, care should always be taken not to over-interpret non-significant findings. In the present study, at injection, no differences were found between the maximum vocalization energy or calls per second for piglets administered 0.3 mL of the local anesthetic vs. sham handled piglets. This result would be in line with our hypothesis that administration of a lower volume of the anesthetic would reduce the acute responses during injection, potentially explained by a lower intra-testicular pressure (26). Yet, a corresponding pattern was not recorded for any other indicator, potentially because of confounding between the dose of anesthetic and volume of drug injected. Performing the injection of 0.3 mL of local anesthetic was also faster than injection of 0.5 mL (35 s vs. 37 s from placement in the bench until removal from the bench). Yet, piglets castrated after administration of 0.3 mL of the local anesthetic per testis did not respond differently from piglets castrated without prior anesthesia in terms of foreleg movements, maximum vocal energy or call proportion, indicating that this volume may not have allowed proper anesthesia of the testes and/or skin. Although not statistically significant, injection of 0.3 mL also led to a 30% increased occurrence of foreleg movements during castration compared to injection of 0.5 mL. Thus, based on the present results, the use of 0.5 mL of procaine-based anesthetic per testis might seem preferable compared to the use of 0.3 mL, as suggested by Courboulay et al. (21). However, this combination of dose/volume (0.5 mL) led to signs of piglet pain at injection, while responses at castration did not differ significantly from those of non-injected castrated piglets in terms of call rate, entropy, and foreleg movements. Concerns regarding the potential of procaine anesthetic injections to fully mitigate piglet pain therefore remain.

In addition, we note that the current recommendations to administer up to 0.5 mL per testis [guidelines from the Danish Veterinary and Food Administration, (22)] are given for piglets up to 7 days of age, regardless of their weight. Considering the large variation in weight in piglets between 2 and 7 days of age, the actual efficacy of the dose of anesthetic when administered to larger piglets (older than the 3–4 day old piglets in our study) may need to be verified. Reversely, relatively small piglets may be administered a dose potentially leading to adverse side-effects, as also suggested by Abendschön et al. (14). In the present study, several piglets of around 1 kg of weight were administered 1.0 mL in total, corresponding to an actual dose of approximately 17.3 mg procaine/kg. In dogs and horses, intravenous administration of similar doses of procaine is considered sub-lethal, leading to behavioral, locomotor and vascular reactions (27). It is, however, unknown whether these effects are applicable to intra-testicular injections. In the context of castration, it is often assumed that most of the drug administered is removed together with the severing of the testes, and therefore that systemic effects remain limited or inexistent. Yet, there is, to our knowledge, a lack of data about the pharmacokinetics of procaine as administered in the testes, as well as on the behavioral and/or physiological impact of the administration of different doses (mg/kg piglet) of procaine in pigs in the context of castration. This matter will be examined in an upcoming study of ours. We also note that no experimental data on dose toxicity, nor general tolerance of procaine in pigs, appear to exist. Thus, future studies are required to examine potential systemic effects.

In the present study, evaluation of the effect of the volume of drug was confounded by dose, reporting the combined effects of physical pressure applied on the testes resulting from the volume of liquid injected (26) and anesthetic efficacy obtained from the dose of anesthetic administered. This design was chosen to reflect practical options, i.e., without requiring alterations to the commercially available drug, while still following recommendations of the DVFA (22). However, further insights on the effect of local anesthesia may be achieved by conducting distinct studies of the efficacy of the dose and impact of the injected volume, for instance using different volumes of saline injections. Similarly, investigation of the effect of the drug volume could have included injections of more than 0.5 mL of drug per testis, in order to test potential benefits in terms of efficacy at castration. However, this practice would not fall within the recommendations given by the DVFA in terms of volume of drug injected, and concerns regarding potential dose-related side-effects would be increased. In addition, with an average testicular weight not exceeding 0.5 gram in 3–4 day old piglets (results not presented), a liquid injection of more than 0.5 mL may represent a considerable intra-testicular pressure, potentially damaging the integrity of the tissue, and creating a counter-productive effect for animal welfare. Yet, these effects remain speculations. While intra-testicular injection of 0.5 mL of a procaine-based local anesthetic was reported not to cause testicular tissue damage in 4–6 day old piglets (28), the impact of the volume of drug injected on the integrity of the testes in piglets as early as 3 day old have not been experimentally studied.

In the present study, the magnitude of the cortisol response was comparable between castrated and sham handled piglets. Thus, this response may reflect an activation of the hypothalamic–pituitary–adrenal (HPA) axis related to stress induced by handling rather than the affective component of pain alone. This suggestion is in line with the on-going debate regarding the interpretation of cortisol responses in terms of specific emotional states such as pain, considering that activation of the HPA axis may occur through handling alone (29, 30). In relation to foreleg movements, we found no evidence for either habituation (fewer responses) or sensitization (more responses) to repeated stays in the bench, indicating potential accumulative effects. It was thus somehow unexpected that the cortisol response to sham handling as one placement in the castration bench did not differ significantly from the response to two placements in the bench, corresponding to a longer total handling duration. However, we cannot exclude that the two manipulations, separated by a five min interval, led to a different temporal curve of the cortisol response. Although the timing of cortisol sampling is within the range previously used to record acute responses to castration in young piglets [e.g., (16, 31)], our study included only one post-castration saliva sample and no unhandled group. Therefore the results cannot be used to identify peak or maximum cortisol response to castration. Acquiring such knowledge would require repeated sampling of saliva, representing an additional stressor, or sampling of blood from catheterized piglets. It is possible that part of the cortisol response was induced by piglets being kept away from the sow and the home pen, rather than as a response to the procedures *per se*. Yet, the use of the heated area with straw and littermates for piglets waiting to be exposed to procedures would be expected to limit these effects. A last possibility could relate to potential ceiling effects in cortisol concentrations, reaching a maximum level with a single manipulation alone. Previous work has considered the relative lack of sensitivity of cortisol as a limitation for its use as an indicator of stress or pain, as cortisol concentrations may be limited by adrenal exhaustion (32, 33). In that perspective, the actual arousal response of piglets to the procedures may be underestimated if considered solely based on the cortisol responses. Further studies investigating the sensitivity of cortisol as an indicator of stress in combination with pain in neonates are warranted.

In conclusion, and in light of the present results, impacts of the needle injections and associated handling should be considered in relation to the use of local anesthetics for piglet castration. As also reported by Leidig et al. (11), our study showed that despite reducing piglets' responses to castration, the use of local anesthetics led to a prolonged duration of the procedures, suggested to be the reason for the increased overall responses in vocalizations and foreleg movements. In the present analyses, we used simple addition of the responses toward injection and castration to illustrate a total response. It is not presently known, though, whether piglet perception of pain and stress is linear or additive. e.g., whether five mild stressors are worse than one severe. This topic warrants further study. Nevertheless, the present study showed that injection in itself led to responses indicative of stress and pain, and suggested that the responses of the piglets to the two procedures combined were comparable, if not greater, than when piglets were castrated without anesthesia. Thus, although the results suggested that injections with 0.5 mL of a procaine-based anesthetic led to stronger pain mitigation, as compared to injections with 0.3 mL, the overall benefit of the procedure for piglet welfare remains arguable. In perspective, while other types of local anesthetics may be considered in the future to improve efficacy, the required additional handling and painful injections, as well as the limited knowledge on potential side-effects of anesthetic drugs in the context of castration of male piglets, remain significant concerns.

## Data availability statement

The original contributions presented in the study are included in the article/Supplementary material, further inquiries can be directed to the corresponding author.

## Ethics statement

The animal study was reviewed and approved by the Danish Animal Experiments Inspectorate. Written informed consent was obtained from the owners for the participation of their animals in this study.

## Author contributions

The study was designed by the project group involving MH, JM, MC, and MK with advice from LF. MK developed the anesthesia injections and trained the herdsman. MC was in charge of the data collection, data processing, and data analysis with supervision from JM, LF, and MH. LF performed the sample size calculations. MC drafted the manuscript. All authors contributed to the article, read, and approved the final manuscript.

## Funding

This study was commissioned and funded by the Danish Veterinary and Food Administration (DVFA) as part of the agreement with Aarhus University on research-based policy support. In addition, Arhus University funded part of the study.

## Conflict of interest

The authors declare that the research was conducted in the absence of any commercial or financial relationships that could be construed as a potential conflict of interest.

## Publisher's note

All claims expressed in this article are solely those of the authors and do not necessarily represent those of their affiliated organizations, or those of the publisher, the editors and the reviewers. Any product that may be evaluated in this article, or claim that may be made by its manufacturer, is not guaranteed or endorsed by the publisher.

## Supplementary material

Supplementary Dataset Dataset used for statistical analysis.

Supplementary Figure S1 The adapted castration bench used during injection, castration and sham handling.

Supplementary Figure S2 Visual representation of the four leg movements recorded during local anesthesia injection, castration, or sham handling while in the castration bench. F, Flexion; K, Kick; E, Extension; B, Blow.

Supplementary Figure S3 Flow diagram of the number of piglet included in the analysis of each type of indicator. Items with slipped corner represent the number of piglet removed at a particular stage and the corresponding reasoning.
