# Supplementary material for: Piglets' acute responses to procaine-based local anesthetic injection and surgical castration: Effects of two volumes of anesthetic
Source: Front Pain Res (Lausanne). 2022 Aug 9;3:943138. doi: 10.3389/fpain.2022.943138 (PMC9395716; doi:10.3389/fpain.2022.943138)
Supplement: Supplementary Figure S1 — The adapted castration bench used during injection, castration and sham handling. [file Image_1.pdf]

S1 Fig. The adapted castration bench used during injection, castration and sham handling.

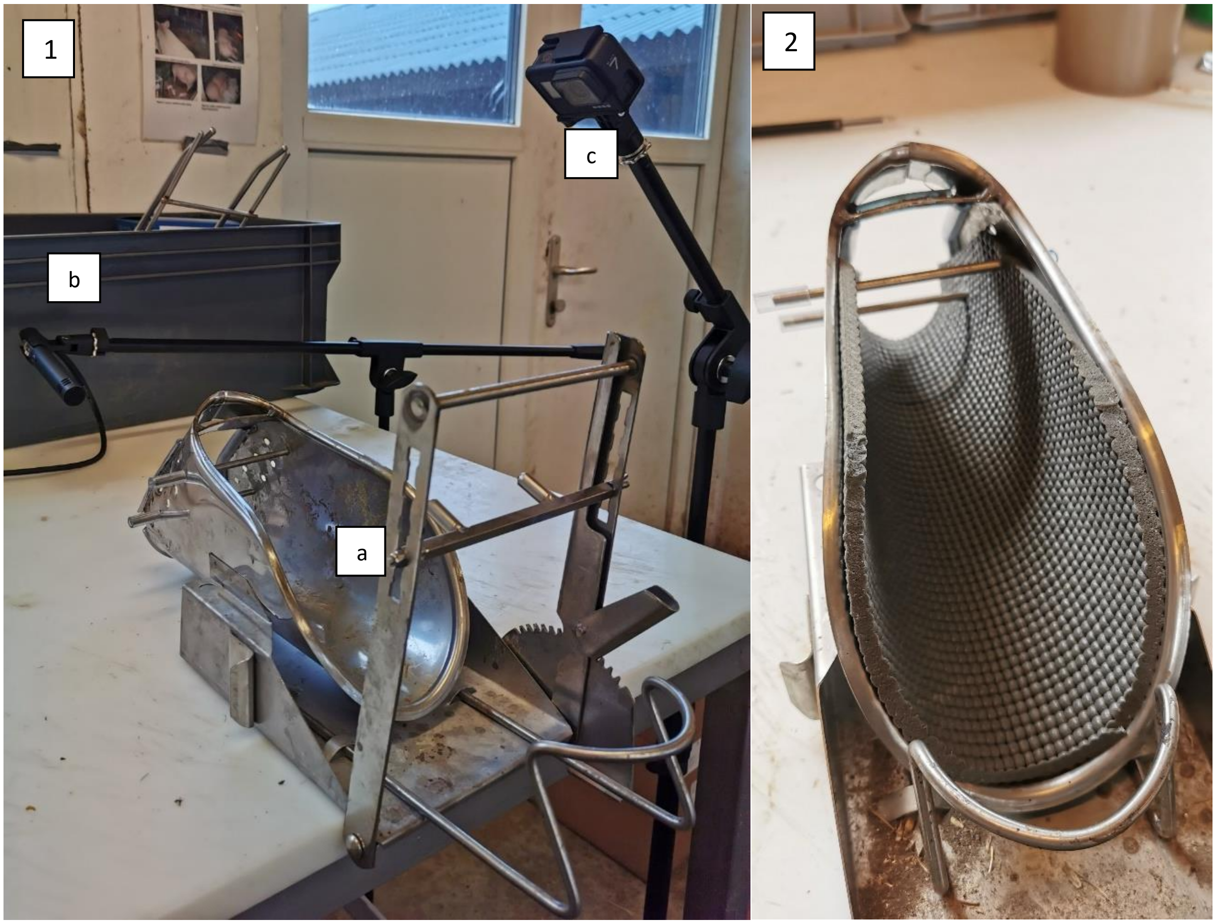

Picture of the bench (1a), with microphone for vocalisation recording (1b), camera for recording of the resistance movements (1c) and soft layer matt ensuring the fitting of each piglet during the interventions (2).
