# Supplementary figures and images for: Piglets' acute responses to procaine-based local anesthetic injection and surgical castration: Effects of two volumes of anesthetic
Source: Front Pain Res (Lausanne). 2022 Aug 9;3:943138. doi: 10.3389/fpain.2022.943138 (PMC9395716; doi:10.3389/fpain.2022.943138)

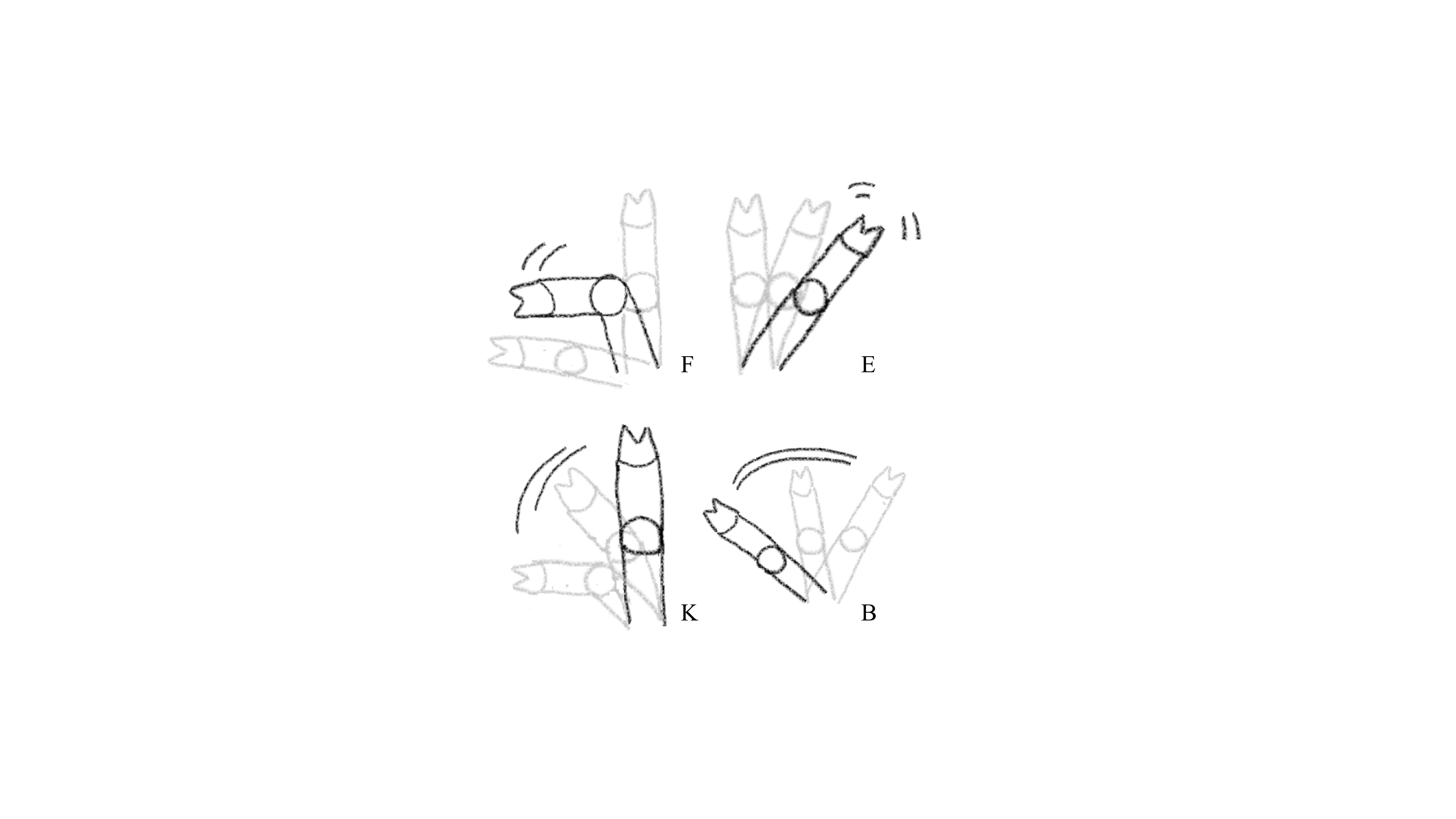

Supplement: Supplementary Figure S2 — Visual representation of the four leg movements recorded during local anesthesia injection, castration, or sham handling while in the castration bench. F, Flexion; K, Kick; E, Extension; B, Blow. [file Image_2.tif]

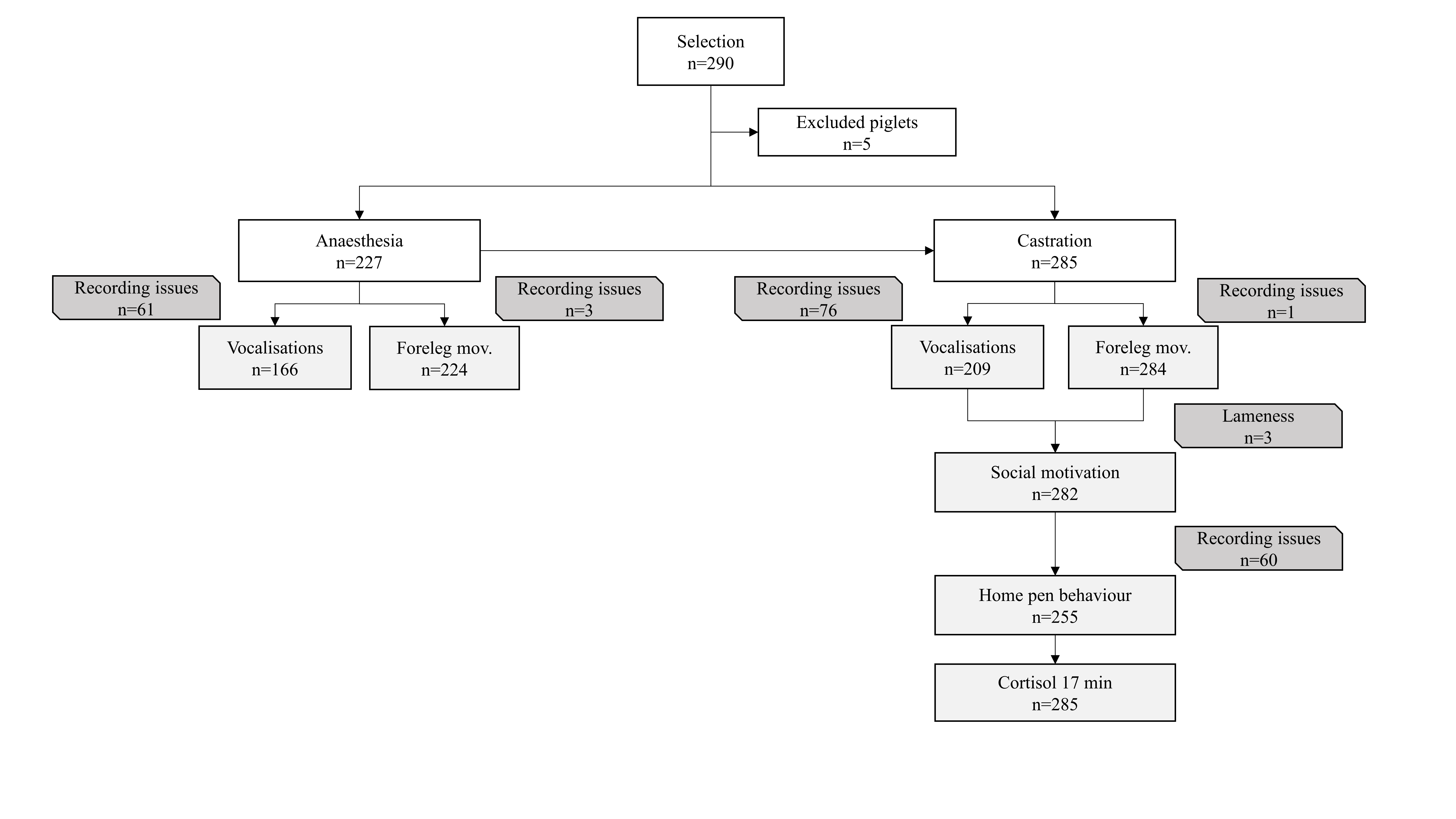

Supplement: Supplementary Figure S3 — Flow diagram of the number of piglet included in the analysis of each type of indicator. Items with slipped corner represent the number of piglet removed at a particular stage and the corresponding reasoning. [file Image_3.tif]
